# Can financial insecurity and condescending treatment explain the higher prevalence of poor self-rated health in women than in men? A population-based cross-sectional study in Sweden

**DOI:** 10.1186/1475-9276-11-50

**Published:** 2012-09-01

**Authors:** Anu Molarius, Fredrik Granström, Inna Feldman, Marina Kalander Blomqvist, Helena Pettersson, Sirkka Elo

**Affiliations:** 1 Västmanland County Council, Competence Centre for Health, 721 89 Västerås and Karlstad University, Karlstad, Sweden; 2 Research & Development Centre,, Sörmland County Council, Eskilstuna, Sweden; 3 Department of Community Medicine, Uppsala County Council, Uppsala, Sweden; 4 Department of Community Medicine, Värmland County Council, Karlstad, Sweden; 5 Department of Community Medicine and Public Health, Örebro County Council, Örebro, Sweden

**Keywords:** Gender, Health inequalities, Self-rated health, Population surveys, Sweden

## Abstract

**Introduction:**

Women have in general poorer self-rated health than men. Both material and psychosocial conditions have been found to be associated with self-rated health. We investigated whether two such factors, financial insecurity and condescending treatment, could explain the difference in self-rated health between women and men.

**Methods:**

The association between the two factors and self-rated health was investigated in a population-based sample of 35,018 respondents. The data were obtained using a postal survey questionnaire sent to a random sample of men and women aged 18-75 years in 2008. The area covers 55 municipalities in central Sweden and the overall response rate was 59%. Multinomial odds ratios for poor self-rated health were calculated adjusting for age, educational level and longstanding illness and in the final model also for financial insecurity and condescending treatment.

**Results:**

The prevalence of poor self-rated health was 7.4% among women and 6.0% among men. Women reported more often financial insecurity and condescending treatment than men did. The odds ratio for poor self-rated health in relation to good self-rated health was 1.29 (95% CI: 1.17-1.42) for women compared to men when adjusted for age, educational level and longstanding illness. The association became, however, statistically non-significant when adjusted for financial insecurity and condescending treatment.

**Conclusion:**

The present findings suggest that women would have as good self-rated health as men if they had similar financial security as men and were not treated in a condescending manner to a larger extent than men. Longitudinal studies are, however, required to confirm this conclusion.

## Introduction

The WHO report on social determinants of health concludes that reducing the health gap between nations and within nations is only possible through addressing gender inequities [1]. Inequity is defined as biases in the conditions of daily living that are systematic, produced by social norms, policies, and practices that tolerate or actually promote unfair distribution of and access to power, wealth, and other necessary social resources [1]. Following this definition gender inequities can be defined as unfair systematic differences between men and women in the conditions of daily living that are shaped by these social structures and processes. In order to reduce the health gap it is therefore important to elucidate possible reasons for gender differences in health.

Self-rated health is a widely used indicator of health and has been found to be a good predictor of morbidity and mortality [2-5]. Large socioeconomic differences have been observed in self-rated health with persons with low socioeconomic status having, in general, poorer self-rated health than persons with high socioeconomic status [6-10]. In most countries women have poorer health than men, which also applies to self-rated health [11-13].

In the literature, several pathways have been studied to elucidate socioeconomic differences in health. Both material conditions [7-9,14] and psychosocial factors [6,10,13,14] have been found to be strongly related with poor self-rated health. In a previous study [12], the strongest association with poor self-rated health was found for condescending treatment (being belittled) when adjusted for the other material and psychosocial factors included in the analysis. Economic hardship, lack of social support, and employment status were also important contributing factors [12]. For material conditions, longitudinal studies have shown that financial insecurity is a strong risk factor for poor self-rated health [14,15].

What comes to psychosocial factors, there is strong evidence that for example perceived discrimination has a negative effect on both physical and mental health [16]. Discrimination can be defined as “the process by which a member, or members, of a socially defined group is, or are, treated differently (especially unfairly) because of his/her/their membership of that group” [17]. The term s*tructural* discrimination refers to the totality of ways in which societies foster discrimination, while the term *interpersonal* discrimination refers to discrimination in practical situations [18].

At the state level in the US, it has been found that women who live in states with more political participation, higher employment and earnings and better economic autonomy have better self-rated health than in states where women’s political and economic circumstances are worse [19]. In general, women tend to have poorer economic resources than men [1]. In Sweden, both self-reported measures [12,20] and objective measures such as income [21] show that women’s economic resources are poorer than men’s. Whereas some types of protecting psychosocial factors such as social support are more prevalent among women than among men [12,13], women report condescending treatment [12] and perceived discrimination [22] more often than men. Our aim was therefore to investigate whether two factors, one material (financial insecurity) and one psychosocial (condescending treatment), could explain the difference in self-rated health between women and men.

## Methods

The present study is based on a cross-sectional postal survey questionnaire sent to a population sample of men and women aged 18-84 years. The area investigated covers 55 municipalities in five counties in central part of Sweden with about one million inhabitants in this age range. The main purpose of the survey was to gather information about health, lifestyle, living conditions and health care use in the adult population. The survey was conducted during March-May 2008. The sampling was random and stratified by gender, age group, county and municipality. The sample size was 68,710 with an overall response rate of 59%. The data collection was completed after two postal reminders. A total of 40,674 persons returned the questionnaire.

The individuals in the sample were informed that responded questionnaires would be linked to the Swedish official registries through the personal identification numbers, to achieve register information on gender, age, geographic area, educational level and country of birth. The respondents thus accepted the linking of registry data by informed consent. The personal identification numbers were deleted directly after the record linkage. Statistics Sweden, the statistical administrative authority in Sweden, carried out the sampling, collected the data, performed the linkage with register data and delivered de-identified data to the county councils. The survey was approved by the boards of the County Councils of Uppsala, Sörmland, Västmanland, Värmland and Örebro. The study was conducted following the ethical principles of the Helsinki declaration and the data are protected by The law of official statistics (2001: 99 6§) and the The law of secrecy (1980: 100 9 kap. 4§). The Ethical Review Act of Sweden (2003:460) at the time of the data collection did not require an approval of an ethics committee since the data are anonymous. The Swedish national education register only includes individuals up to 75 years of age and therefore the analyses in the present study were limited to age group 18-75 years (N = 36,085).

Self-rated health was assessed with the question “How do you rate your general health?” with the options “very good”, “good”, “neither good nor poor”, “poor” and “very poor”. For the analysis the categories “very good” and “good” were combined, as were the categories “poor” and “very poor”.

Financial insecurity was assessed by asking whether the respondent was able to acquire an amount of 20,000 Swedish crowns in a week in case of unexpected expenses (yes/no).

Condescending treatment was assessed with the question “Have you during the past three months at any time felt that you have been treated in a condescending manner by anyone?”. The answer categories were never, once or twice, and several times.

Because educational level and longstanding illness are associated with self-rated health and differ between men and women, they were treated as possible confounding variables in the analysis. Educational level was obtained through record linkage to information from a national education register and was categorized into three classes: low (elementary school), medium (upper secondary school), and high (at least 3 years of university or corresponding education). We investigated self-rated health among women compared to men within categories of longstanding illness because we wanted to take into account that persons with longstanding illness may have more financial insecurity and may interpret social situations as more negative than persons with no longstanding illness. Longstanding illness was assessed asking whether the respondent had any longstanding illness (longer than 6 months), permanent ailment or disability (yes/no).

Age was grouped into four age groups (18-34, 35-49, 50-64 and 65-75 years) in the analysis. Subjects with missing data for the studied factors were excluded from the analysis. The proportion of missing data was 1.9% for financial insecurity and 2.3% for condescending treatment. The total number of respondents included in the present study was 35,018.

Differences in the prevalence of risk factors and self-rated health between women and men were tested with chi-squared statistics. A multinomial logistic regression analysis was performed. The results are reported as odds ratios (OR) and 95 percent confidence intervals (95% CI) for having poor self-rated health among women compared with men. Similar odds ratios were calculated for neither good nor poor self-rated health. The category of good self-rated health was the constant category. First, we calculated bivariate odds ratios between the risk factors and self-rated health. In the second model, the odds ratio for gender was adjusted only for age group and for the covariates educational level and longstanding illness. Next we added the two factors financial insecurity and condescending treatment separately. Last, we added both financial insecurity and condescending treatment simultaneously into the model. Due to exclusion of subjects with missing data the number of respondents in the final model was 32,455. To test whether the results differ between age groups or by educational level, similar analyses were carried out within age groups 18-49 years and 50-75 years as well as within high and low educational levels.

## Results

Women were somewhat younger and had higher educational level than men (Table 1). The difference in prevalence of longstanding illness was rather small. Women had a higher prevalence of financial insecurity and condescending treatment during the last three months than men. The overall prevalence of poor self-rated health was 7.4% among women and 6.0% among men. The difference between women and men in neither good nor poor self-rated health was smaller. The differences in the distributions of all these variables were statistically significant between women and men.

**Table 1:** Number of subjects, mean age and other characteristics of the study population (crude data)

	**Women**	**Men**	**Total**	**p-value for difference between women and men**
N	19 330	15 688	35 018	
Age, mean (SD)	49.1 (16.4)	52.0 (16.1)	50.5 (16.3)	<.001
High educational level (%)	27.3	20.2	24.2	<.001
Longstanding illness (%)	30.9	28.6	29.9	<.001
Financial insecurity (%)	24.8	15.8	20.8	<.001
Treated in a condescending manner during the last 3 months				<.001
· once or twice (%)	23.3	13.5	18.9	
· several times (%)	4.0	1.9	3.1	
Self-rated health				<.001
· neither good nor poor (%)	19.8	20.5	20.1	
· poor (%)	7.4	6.0	6.8	

Table 2 gives the bivariate (unadjusted) odds ratios for gender, financial insecurity and condescending treatment (Model 1). There was a statistically significant difference between men and women in odds for poor self-rated health but not for neither good nor poor self-rated health. Financial insecurity and condescending treatment were strongly associated with both poor and neither good nor poor self-rated health.

**Table 2:** Odds ratios (and 95% confidence intervals) for poor and neither good nor poor self-rated health

		**Model 1**	**Model 2**	**Model 3**	**Model 4**	**Model 5**
		**(bivariate)**		**(2 + financial insecurity)**	**(2 + condescending treatment)**	**(2+ financial insecurity and condescending treatment)**
**Poor self-rated health**						
Gender	Men	1	1	1	1	1
	Women	1.25 (1.15, 1.37)	1.29 (1.17,1.42)	1.18 (1.07, 1.30)	1.17 (1.06, 1.30)	1.08 (0.98, 1.20)
Financial insecurity	No	1		1		1
	Yes	3.34 (3.05, 3.65)		3.01 (2.71, 3.35)		2.80 (2.51, 3.12)
Condescending treatment during the last 3 months	Never	1			1	1
	Once or twice	2.01 (1.82, 2.22)			2.23 (1.98, 2.51)	2.09 (1.86, 2.36)
	Several times	6.40 (5.44, 7.52)			5.71 (4.67, 6.97)	4.85 (3.95, 5.95)
**Neither good nor poor self-rated health**						
Gender	Men	1	1	1	1	1
	Women	0.98 (0.93, 1.03)	1.05 (0.99,1.12)	1.00 (0.94, 1.06)	0.99 (0.93, 1.05)	0.94 (0.88, 1.00)
Financial insecurity	No	1		1		1
	Yes	1.91 (1.80, 2.03)		1.91 (1.77, 2.05)		1.84 (1.71, 1.98)
Condescending treatment during the last 3 months	Never	1			1	1
	Once or twice	1.37 (1.29, 1.47)			1.74 (1.61, 1.87)	1.69 (1.56, 1.82)
	Several times	2.19 (1.89, 2.53)			2.47 (2.10, 2.92)	2.29 (1.93, 2.71)

Models 2-5 are adjusted for age group, educational level and longstanding illness.

The odds ratio for poor self-rated health in relation to good self-rated health was 1.29 for women compared to men when adjusted for age group, educational level and longstanding illness (Model 2 in Table 2). The association attenuated but was still statistically significant when adjusted for financial insecurity or condescending treatment separately. The association became, however, statistically non-significant when adjusted for both financial insecurity and condescending treatment (Model 5 in Table 2). The odds for neither good nor poor self-rated health - when adjusted for age group, educational level and longstanding illness - did not differ by gender.

We repeated the regression analyses in two separate age groups 18-49 and 50-75 years old. The odds ratio for poor self-rated health in women compared to men when adjusted for age group, educational level and longstanding illness was somewhat higher (OR = 1.45 95% CI: 1.22, 1.71) in the younger age group than in the older age group (OR = 1.25 95% CI: 1.11, 1.41). The confidence intervals were, however, overlapping indicating that the difference in these odds ratios between the age groups was not statistically significant. In addition, we repeated the analyses within groups with low and high educational level but the difference in odds ratios for poor self-rated health for women compared to men between the educational levels was not statistically significant (data not shown).

## Discussion

The WHO report on social determinants of health concludes that reducing the health gap is only possible through addressing gender inequities [1]. Empowerment of women is considered as a key to achieving fair distribution of health. In this cross-sectional study, the difference in poor self-rated health between men and women could be explained by a higher prevalence of financial insecurity and experiences of condescending treatment among women.

In previous studies, social relations in the form of social capital, support, and networks have been found to be important determinants of self-rated health [6,10,13]. It is assumed that the quality of social interaction results in psychological reactions, which in turn affect health. In previous studies perceived discrimination has been found to be a strong determinant of both physical and mental health [16]. We did not measure perceived discrimination directly, but it can be assumed that if a person has been treated several times in a condescending manner this can be experienced as discrimination. In the literature, this type of discrimination is often referred to as everyday interpersonal discrimination i.e. being treated badly in everyday situations [23]. Therefore there is probably a high correlation between perceived discrimination and condescending treatment, but this, of course, has to be confirmed in subsequent studies.

Psychosocial pathways are, however, likely to only partly explain social differences in self-rated health. Pathways based on material indicators, such as economic hardship and financial insecurity, are also important [7-9,14]. In our study, adjusting only for condescending treatment or for financial insecurity was not enough to explain the higher prevalence of poor self-rated health among women. But when the two factors were adjusted for simultaneously, the difference in poor self-rated health became statistically non-significant. Both factors were also strongly associated with poor self-rated health. It should be noted, however, that the original difference in self-rated health between women and men was not that large.

Women had higher educational level than men which is in line with the official statistics [21]. In spite of this they had a higher level of financial insecurity than men. This suggests that education does not “pay-off” as much among women as it does among men. In Sweden, women’s incomes are in general 75% of men’s incomes. Even though women participate in the working force almost to the same extent as men do, the labor market is strongly segregated with women working more often in occupations with lower salaries [21]. In addition, women with small children often work part-time and women spend, in general, more time in domestic work than men do whereas having children does not affect the working time of men [21]. The fact that women have higher educational level than men but have worse economic situation can be interpreted as a structural level, unobserved discrimination [18]. It is against the WHO report’s intention of equality in terms of equitable distribution of power, money and resources and the structural drivers of those conditions of daily life [1]. It also suggests that promoting high education may not be sufficient to improve the health of women if there are other structural mechanisms that counteract its effect.

The response rate in our study was not very high (59%) but similar to other population based studies in Sweden [20,22]. The response rate was lower among younger than among older subjects and in men compared with women. The respondents had also somewhat higher educational level than the general population of the same age. It is possible that men with poor health are underrepresented among the respondents and therefore the true difference in self-rated health between men and women may be smaller. Also the financial situation of men can be overestimated. On the other hand, the poorer health and economic situation of women in Sweden is well documented [21]. The level of condescending treatment among women can be overestimated in the case of response bias. But current evidence shows, on the contrary, that women tend rather to underreport than to over report discriminatory experiences [24]. This can be explained by the notion that subordinate groups are more likely to deny or underreport discriminatory experiences because they may internalize negative attitudes by accepting the dominant culture’s values and role in society [18]. Based on these arguments it is unlikely that response bias would entirely explain the results obtained.

A major limitation of the present study is that it based on cross-sectional data. It is therefore not possible to draw conclusions about which factors are causes and which are effects of poor self-rated health. Persons who have experiences of condescending treatment may have higher risk of poor health, but persons with poor health may be treated condescendingly due to their health status. Also, the relationship between financial insecurity and health can be reverse, if persons with poor health run into financial difficulties, for example due to receiving sickness compensation instead of salary. We took into account the possible reverse effect by adjusting for longstanding illness. Adjusting for longstanding illness did not, however, affect remarkably the estimated odds ratios suggesting that either this reverse effect was not substantial or that we did not capture it very well by adjusting for longstanding illness. In any case, prospective studies have shown that both financial insecurity [14,15] and poor social relations [13,14] as well as perceived discrimination [16] have independent causal effects on self-rated health.

A limitation of the study is also that both the risk factors and the outcome were self-reported. For self-rated health this is the only option and it has been shown to be a good measure of health [2-5]. What comes to financial insecurity, there is some evidence that self-reported measures of economic difficulties are more strongly related to health than measures based on objective economic situation such as low income [20]. Previous studies have used different measures of financial insecurity [14,15]. Our measure was similar to one of two questions used to measure self-reported economic difficulties in the Swedish study [20]. Self-reported condescending treatment has previously been shown to be strongly associated with poor self-rated health [12] and mental health symptoms [25]. Similar results have been reported for the association between self-reported experiences of discrimination and psychological distress [22]. There was also a dose–response relationship between condescending treatment and self-rated health, which has also been reported for perceived discrimination [26]. Experiences of discrimination are usually measured through self-reports and different studies have often used different measures of perceived discrimination since there has been a lack of validated instruments that could have been used in large scale epidemiologic studies [16,18].

We adjusted for educational level and longstanding illness in the analyses, because these factors are associated with self-rated health and differ between women and men. There was, however, a statistically significant difference between women and men in self-rated health even when educational level and longstanding illness were taken into account. This difference was also similar among younger and older subjects. In addition, the questions on financial insecurity and condescending treatment can have different meaning for persons with different educational levels. Persons with a longstanding illness such as depression, on the other hand, can be more likely to interpret social situations as negative. Since these factors were accounted for, our results cannot be explained to any larger extent by differences in educational level or longstanding illness between women and men. We also investigated the difference in self-rated health between women and men and the effect of financial insecurity and condescending treatment within different educational levels but the results were fairly similar indicating that the results do not differ by educational level.

In our study, it was the higher prevalence of financial insecurity and experiences of condescending treatment among women that explained the difference in poor self-rated health between women and men. It is, however, possible that similar results would have been obtained using some other measures of material conditions and psychosocial factors closely related to these two indicators. The strength of our study is that it is based on a very large general population and includes a wide age range. The robustness of the results irrespective of age group and educational level further strengthens the implications of the results.

The difference in self-rated health between women and men was not very large, but similar to several other West-European countries [11]. Even in Sweden where the opportunities for men and women in society are relatively equal, this difference exists and is relatively consistent between age groups and educational levels. The findings of present study imply that gender differences in health can be explained by material conditions and psychosocial factors that can be measured at individual level but are produced at structural and interpersonal level through mechanisms of different types of discrimination [18]. This is important since women comprise half of the population and addressing these differences has therefore a large effect on health and on health inequities in the general population [1,27].

## Conclusion

While a cross-sectional study does not allow definite conclusions as to which factors are determinants and which are consequences of poor self-rated health, the present findings suggest that women would have as good self-rated health as men if they had similar financial security as men and were not treated in a condescending manner to a larger extent than men. This conclusion needs, however, to be confirmed by results from longitudinal studies.

## Authors’ contributions

All authors contributed to originating the idea. FG and AM analyzed the data and AM wrote the manuscript draft. All authors contributed to interpretation of the results and critically revised the manuscript. All authors approved the final version of the manuscript.

## Competing of interest

The authors declare that they have no conflict of interest.
